# The Use Of Oral Ranolazine To Convert New Or Paroxysmal Atrial Fibrillation: A Review Of Experience With Implications For Possible "Pill In The Pocket" Approach To Atrial Fibrillation

**Published:** 2009-09-01

**Authors:** David K Murdock, Mary Kersten, Jeff Kaliebe, German Larrain

**Affiliations:** The Aspirus Heart and Vascular Institute, Cardiovascular Associates of Northern Wisconsin and The CaRE Foundation, Wausau Wisconsin USA

**Keywords:** atrial fibrillation, ranolazine, conversion, anti-rhythmic therapy, anti-rhythmic agents

## Abstract

**Background:**

Atrial fibrillation (AF) is the most common arrhythmia requiring treatment. High dose oral anti-arrhythmics may cardiovert some paroxysmal AF. This "pill in pocket" approach has allowed patients to treat themselves on an as needed basis. Pro-arrhythmic concerns have limited the usefulness of this approach to patients without structural heart disease.  Ranolazine is an anti-anginal agent, which inhibits abnormal late Na+ channel currents in cardiomyocytes and decreases sodium-calcium overload. Ranolazine is a potent inhibitor of after-depolarizations, which have been implicated in the initiation and propagation of AF. Because ranolazine has no known pro-arrhythmic effects, it could be useful as a safe "pill in the pocket" agent if it were effective in converting AF. We describe our experience using oral ranolazine to convert new or paroxysmal AF.

**Methods:**

2000 mg of ranolazine were administered to 18 patients with new (11 patients) or paroxysmal (7 patients) AF of at least 3, but not greater than 48 hours duration. Most patients (14) were in the hospital at the time ranolazine was administered. Age, sex, echocardiographic data, associated health conditions and structural heart disease were recorded. Successful conversion was defined as restoring sinus rhythm within 6 hours of ranolazine administration.

**Results:**

All but 1 patient had some form of structural heart disease and all but 2 patients had left atrial enlargement. Thirteen of 18 patients converted to sinus rhythm. No pro-arrhythmic effects, hemodynamic instability, adverse rate effects, or perceived intolerance (other than constipation) were noted. The 72% conversion rate was comparable to other reported "pill in the pocket" protocols.

**Conclusions:**

High dose oral ranolazine shows utility as a possible safe agent to convert new or paroxysmal AF. Lack of blinded controls and small numbers limits the power of this observation.

## Introduction

Atrial fibrillation (AF) is the most common arrhythmia requiring treatment  [1,2]. Transthoracic electrical cardioversion has remained the most effective method for terminating AF since its introduction over 4 decades ago [3]. Alternatively, some patients may convert to normal sinus rhythm with anti-arrhythmic therapy [4-13]. Pro-arrhythmic concerns and the lack of a safe anti-arrhythmic agent have limited the usefulness of anti-arrhythmic therapy in the un-monitored setting [6,8,9,14,15]. In properly chosen patients high dose oral anti-arrhythmic agents may effectively cardiovert some patients with paroxysmal AF [10-,13]. In this approach, up to 100% of the normal daily dose of propafenone or flecainide is given as a single oral dose to patients without structural heart disease [10-,13]. This "pill in the pocket" approach has allowed these patients to effectively treat themselves on an as needed basis when AF occurs without the need to immediately seek medical attention or use anti-arrhythmic therapy on a chronic basis.

In 1998, Haissaguerre et al demonstrated that atrial fibrillation (AF) may originate and be perpetuated from ectopic activity originating at the junction of the left atrium and pulmonary veins [16]. The electrophysiologic mechanisms responsible for the abnormal impulse activity have been the source of several investigations [17-23]. Recently it has been determined that triggered activity may be particularly important.

Ranolazine is an anti-anginal agent, which inhibits normal and abnormal late Na+ channel current in the ventricle and peak Na+ channel current in the atrium [24-26]. By this inhibition, it affects intracellular calcium handling producing an energy sparing effect [24]. Ranolazine has also been shown to be a potent inhibitor of after depolarizations produced by a number of mechanisms [25-28]. As such, it could prove to be particularly useful in the treatment of AF. Indeed, in the holter monitor data from the MERLIN trial, ranolazine was associated with a reduction in a number or several arrhythmias, including new episodes of AF [29,30]. We have extended these observations to show that ranolazine can be successfully employed as an anti-arrhythmic agent and can be particularity useful in atrial fibrillation  [31,32].

Since ranolazine is devoid of known pro-arrythmic effects and is well tolerated, it could prove to be an ideal agent for the "pill in the pocket" approach to AF if it were effective in converting patients with paroxysmal AF to sinus rhythm. The purpose of this report is to review our experience using single dose oral ranolazine as a means to facilitate conversion of AF to sinus rhythm with the possible consequence of a "pill in the pocket" approach to some AF using ranolazine. This was a retrospective analysis of our experience using ranolazine for this purpose. Institutional review board approval is not required for retrospective chart review.

## Study Population

Eighteen patients with a known duration of AF of greater than 3 hours but less than 48 hours agreed to try oral ranolazine to convert their atrial fibrillation. Patients were informed that this was an "off label" use of ranolazine and its ability to convert them to sinus rhythm was unknown. The age, sex, left ventricular ejection fraction (%), left atrial diameter (mm) and volume index (ml/m^²^) by echocardiography, and associated structural heart, and other health conditions were determined for most patients. The history of the AF problem (first recognized episode or paroxysmal) was also determined for each patient. Finally, the location of the first ranolazine treatment (home, office, hospital) was determined.

The treatment with ranolazine consisted of the administration of 2000 mg of ranolazine as a single oral dose. This dose was chosen as it consisted of 100% of the usual maximal recommended daily dose of this agent, similar to the dosing rationale employed with high dose propafenone or flecainide [10-13]. The treatment was considered successful if the interval between administration of the drug and conversion to sinus rhythm was six hours or less and there were no observable side effects, such as symptomatic hypotension (systolic blood pressure,  80 mm Hg), symptomatic bradycardia after restoration of sinus rhythm, dyspnea, presyncope, syncope, or conversion to atrial flutter or atrial tachycardia [11].

Patients were excluded from consideration of this approach if they had a long QT interval, were on any other anti-arrhythmic agent other than beta or calcium channel blockers, had a history of second- or third-degree atrioventricular block, bradycardia-tachycardia syndrome (resting heart rate,  50 beats per minute).

Four patients with a history of paroxysmal AF were given the opportunity of using this approach for recurrent future AF episodes on a "pill in the pocket" basis.  Each was instructed to take the drug at least 10 minutes after any subsequent onset of palpitations. After the ingestion of the drug, a resting state (in a supine or sitting position) was recommended until the palpitations had stopped or at least six hours had passed [11]. These patients were contacted periodically to see if they continued to have episodes of AF and how they responded to "pill in the pocket" ranolazine. Patients were advised to contact the emergency room or our office if their palpitations had not ceased within 12 hours of ingestion of the drug, or if they had symptoms that had not occurred during previous arrhythmic episodes (e.g., intolerable palpitations, dyspnea, syncope, or presyncope).

## Results

Fourteen patients were in the hospital when AF occurred. In these patients, AF was not present at the time of hospitalization but occurred during the course of treatment for other issues. One hospitalized patient was recruited immediately after he had failed electrical cardioversion for new AF. In 2 patients with a history of paroxysmal AF, the AF occurred in the outpatient setting and the patients called our facility with recurrent palpitations. These patients were brought to the office for treatment where the AF was confirmed by an electrocardiogram. Two patients were at home when ranolazine was administered, including the index case to be discussed in more detail.

In 11 patients, the AF was their first recognized episode of AF. Seven others had a history of paroxysmal AF. Each patient was aware of their arrhythmia because of palpitations but were hemodynamically stable  (i.e., without symptoms such as dyspnea, presyncope, or syncope). Table 1 describes the clinical characteristics of the patients in which oral ranolazine was used to attempt to convert AF and the setting initially employed. Note the wide spectrum of associated medical conditions and structural heart disease. Fourteen of the 16 patients in which echocardiographic data was available, had left atrial enlargement as measured by an elevated left atrial volume index exceeding 26 ml/m^²^. Although most of the patients were monitored at the time of first use, 2 patients were in the office during first dose administration and 2 patients were at home.

Table 2 outlines the results of oral ranolazine therapy and current status of the patients. Thirteen of the 18 patients responded to this approach. Of the four patients who used it on an out patient "pill in the pocket" basis, 1 failed therapy and is now in chronic AF having also failed sotalol and dofetilide, 2 continue to use ranolazine as needed for AF episodes and the other returned to chronic ranolazine usage to prevent episodes of AF after using it successfully as a converting agents a few times times. The details of this patient and the significance to this study bear documentation:  Patient 1 (Table 1 and 2) had a history of difficult to control AF, which was originally classified as persistent AF. He had promptly failed both sotalol and dofetilide within days with rapid relapse into AF after cardioversion. Using ranolazine as an anti-arrhythmic agent, this patient was electrically cardioverted to sinus rhythm and remained in sinus rhythm for nearly 1 year on chronic ranolazine (1000mg twice a day)  [32]. Because of cost issues, this patient stopped ranolazine without informing us. Within a short period of time he had developed recurrent AF manifest as recurrent palpitations similar to what had marked his AF symptoms in the past. This prompted him to resume his ranolazine by taking an "extra pill" (2000 mg). He did this a few times over the course of a couple of months before he informed us of this: each time using ranolazine as a conversion medication only. On each occasion 2000 mg of ranolazine would restore sinus rhythm within a few hours of the onset of his typical palpitations where as in the past only electrical cardioversion would restore sinus rhythm. Having repeatedly relapsed after stopping ranolazine, this patient has elected to resume chronic ranolazine therapy rather than continue this "pill in the pocket" approach. Ranolazine has continued to maintain him in sinus rhythm.

Ranolazine was very well tolerated in this setting. No patient experienced any cardiovascular side effects or worsening of AF symptoms. One patient, who continues to use it on a "pill in the pocket" basis notes problems with constipation for approximately 36 hours each time he uses this approach.

## Discussion

We found that 2000 mg of oral ranolazine, when administered as a single oral dose for paroxysmal or new onset AF was associated with a conversion rate to sinus rhythm in 13 of 18 patients (72%) within 6 hours of dose administration. The conversion rate we observed by six hours with ranolazine was similar to 6 to 8 hour conversion rate previously reported with high dose oral "Pill in the Pocket" propafenone or flecainide [10-13] and higher than the 39% placebo 8 hour conversion rate noted by Capucci et al [13]. This dose of ranolazine was not associated with adverse cardiovascular side effects and was very well tolerated. In none of those patients was oral ranolazine associated with any worsening of the symptom of AF prior to conversion.

Our results should not seem surprising. Ranolazine has already been shown to suppress clinical episodes of AF [29,30,32]. In a canine model, ranolazine exhibits powerful use dependent effects on atrial refractory periods, which should give it anti-fibrillatory effects [26]. Additionally, ranolazine is a powerful inhibitor of trigged activity which may be an important mechanism underlying to initiation and potentiation of AF [19-23,25-28]. Finally the time course of the observation is consistent with our prior experience where we reported that ranolazine begins to have significant anti-arrhythmic effects within a couple of hours of administration [31].

## Limitations

The small number of patients in our report makes it essential that these observations be confirmed. Additionally, this was a real life experience with ranolazine. Like all real life clinical decision making regarding anti-arrhythmic therapy, we gauged the effectiveness of ranolazine based the observed clinical response. It is possible that the amount of AF in the 2 patients who continue to use ranolazine on a "pill in the pocket basis" is underestimated due to the occurrence of asymptomatic AF. Because it was not placebo controlled we cannot rule out the possibility that some of these patients would have converted spontaneously within the 6 hours usually observed. Indeed it seems very likely that some would have [10-13]. Larger number of patients and appropriate blinding are clearly indicated. This observation serves as a useful pilot study demonstrating the feasibility of this approach.

## In summary

Ranolazine shows promise as an anti-arrhythmic agent for AF and may be useful to facilitate conversion of AF using a "Pill in the Pocket" approach. Given the apparent electrophysiologic safety of ranolazine and the ability to use it in patients with structure heart disease, such an approach could have enormous economic implications. Further investigations are warranted to explore this novel use for this medication.

## Figures and Tables

**Table 1:**
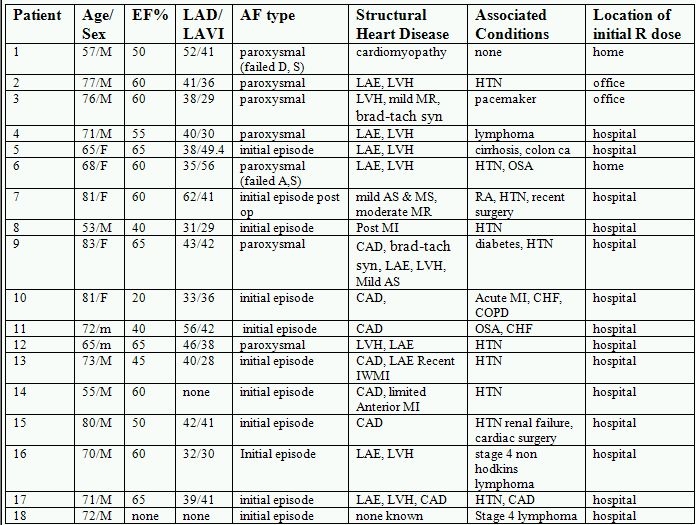
Clinical Characteristics of AF patients

AS: aortic stenosis, brad-tach syn: bradycardia-tachycardia  syndrome, CAD: coronary artery disease, CHF: congestive heart failure, COPD: chronic obstructive pulmonary disease, EF: ejection fraction, D: dofetilide, HTN: hypertension, LAD: left atrial diameter (mm), IWMI: inferior wall myocardial infarction, LAE: left atrial enlargement, LAVI: left atrial volume index (ml/m²), LVH: left ventricular hypertrophy, MI: myocardial infarction, MS: mitral stenosis, MR: mitral insufficiency,  OSA: obstructive sleep apnea, R: ranolazine, RA: rheumatoid arthritis, RHD: rheumatic heart disease. S:sotalol.

**Table 2:**
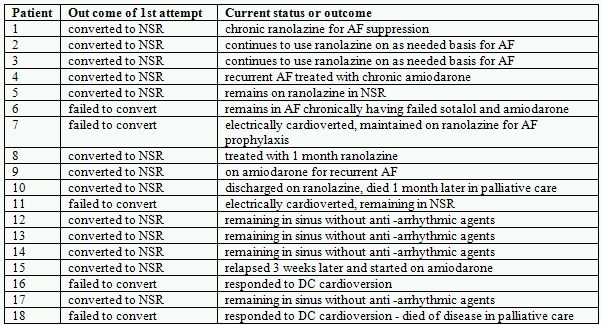
Results of Oral Ranolazine on Atrial Fibrillation

AF: atrial fibrillation, DC Direct current, NSR: normal sinus rhythm
